# Interpreting the meaning of changes in hippocampal volume associated with vestibular loss

**DOI:** 10.3389/fnint.2023.1254972

**Published:** 2023-08-07

**Authors:** Paul F. Smith

**Affiliations:** ^1^Department of Pharmacology and Toxicology, Brain Health Research Centre, School of Biomedical Sciences, University of Otago, Dunedin, New Zealand; ^2^The Brain Research New Zealand Centre of Research Excellence, Eisdell Moore Centre for Hearing and Balance Research, University of Auckland, Auckland, New Zealand

**Keywords:** vestibular dysfunction, hearing loss, cognitive, spatial memory, hippocampus, dementia

## Abstract

Many studies have documented cognitive deficits, especially spatial cognitive deficits, in patients with some form of vestibular loss. Almost 20 years ago, hippocampal (HPC) atrophy was reported to be correlated with spatial memory deficits in such patients and the idea has gradually emerged that HPC atrophy may be causally responsible for the cognitive deficits. However, the results of studies of HPC volume following vestibular loss have not always been consistent, and a number of studies have reported no evidence of HPC atrophy. This paper argues that HPC atrophy, if it does occur following vestibular loss, may not be directly, causally responsible for the cognitive deficits, and that it is more likely that rapid functional changes in the HPC are responsible, due to the interruption of the transmission of vestibular information to the HPC. The argument presented here rests on 3 tranches of evidence: (1) Cognitive deficits have been observed in humans even in the absence of HPC atrophy; (2) HPC atrophy has not been reported in animal studies following vestibular loss, despite cognitive deficits; and (3) Animal studies have shown that the interruption of the transmission of vestibular information to the HPC has immediate consequences for HPC place cells, far too quickly to be explained by HPC atrophy. It is possible that HPC atrophy, when it does occur, is related to the longer-term consquences of living with vestibular loss, which are likely to increase circulating cortisol.

## Introduction

During the last 2 decades, evidence has gradually emerged to suggest that, in addition to its effects on the vestibulo-ocular and vestibulo-spinal reflexes, loss of vestibular function has adverse effects on cognition, especially cognitive processes related to spatial information (examples of recent studies: Deroualle et al., 2019; Dobbels et al., 2019, 2020; Liu et al., 2019; Smith L. et al., 2019; Ayar et al., 2020; Bigelow et al., 2020; Bosmans et al., 2020; Guidetti et al., 2020; Lacroix et al., 2020; Liao et al., 2020; Pineault et al., 2020; Dordevic et al., 2021; Elyoseph et al., 2023; Obermann et al., 2023). Although some of this evidence has not controlled for the potential effects of concurrent hearing loss, many recent studies have, and it has become obvious that both hearing loss and vestibular loss contribute to cognitive dysfunction, with loss of function in both sensory systems exacerbating the situation (see Smith, 2022a,b for a recent discussion).

The presentation of spatial cognitive deficits following vestibular dysfunction naturally implicates the hippocampus (HPC), since it is known to be important for spatial cognition (see Ambrogioni and Ólafsdóttir, 2023, for a recent review). Indeed, through the vestibular nucleus and presumably also the cerebellum, vestibular information is transmitted to the HPC (see Hitier et al., 2014 for a review; see Hitier et al., 2021 for a recent example). Furthermore, inactivation of the vestibular system, through bilateral intratympanic injection of tetrodotoxin (TTX) or bilateral surgical lesions of the vestibular system, results in a dysfunction of HPC place cells (Stackman et al., 2002; Russell et al., 2003), HPC theta rhythm (Russell et al., 2006; Neo et al., 2012) and entorhinal cortex theta rhythm (Jacob et al., 2014). Brandt et al. (2005) originally reported that bilateral vestibular loss in humans was associated with a bilateral decrease in the volume of the HPC of about 17%. By contrast with the functional effects on the HPC, the effects of vestibular loss on HPC volume have been inconsistent, and some researchers have suggested that the original findings of Brandt et al. (2005) were due to the fact that the patients had Neurofibromatosis Type 2 (NF2) (Cutfield et al., 2014). Nonetheless, other studies have also reported HPC atrophy in different patient cohorts (see Table 1). However, the argument of this paper is that the effects of vestibular loss on HPC function are likely to be immediate and may not be related to changes in HPC volume, except over the longer term. This paper will be confined to studies of HPC volume in humans (as opposed to activity or connectivity) or studies closely related, in which vestibular function was specifically tested (as opposed to balance related to postural instability). The studies reviewed were chosen using an NIH PubMed search between 2000 and July 19, 2023, and the search words: “vestibular and hippocampal volume”; “vestibular and hippocampus”; and “vestibular loss and hippocampus.” Only studies in humans and reported in English were included. To be included, a study had to include a control group. Due to the small number of studies, no minimum sample size was specified.

**TABLE 1:** Studies that have quantified hippocampal volume in humans associated with some form of vestibular loss.

References	Type of vestibular loss	N(M/F)	Age and duration	Result for HPC volume
	Unilateral or bilateral vestibular loss (UVL or BVL)			
Brandt et al., 2005	BVL	10 (6, 4) 10 age, sex matched controls	Mean 38 years, 5–10 years	Bilateral atrophy
Hüfner et al., 2007	UVL	8 (4, 4) Left UVL 8 (4, 4) Right UVL 16 age, sex-matched controls	Patients: 47–64 years, 5–13 years Controls: mean, 57.4 years	No change
Cutfield et al., 2014	BVL	12 (6, 6) 15 controls	Patients: mean, 51, >9 months Controls: 46 years of age	No change
Göttlich et al., 2016	Incomplete BVL	27 (16, 11) 29 (16, 13) age and sex-matched controls	Patients: mean 69.2 years, 3 months to 20 years Controls: mean 64.8 years	Bilateral decrease in gray matter in CA3
Dordevic et al., 2021	UVL or BVL	15 (11, 4) 15 controls (11, 4)	Patients: mean, 56.8 years, >6 months controls: 57.6 years	No change
Schöne et al., 2022	UVL or BVL	55 patients BVL: (14, 5) Chronic UVL: (15, 6) Acute UVL: (10, 5) Controls (22, 17)	BVL: mean 53.18 years, mean 14.68 years Chronic UVL: mean, 56.52 years, 8.66 years Acute UVL: mean, 47.35 years, mean 21.47 years Controls: mean 52.07 years	Decrease in right subiculum in UVL, but no change in total or in BVL
Kremmyda et al., 2016	Partial BVL	15 (9, 6) 15 controls (9, 6), age and sex-matched	Patients: mean, 63.6 years, mean, 13.6 years Controls: mean, 63.6 years	Region-specific decrease in bilateral hippocampus
Lee et al., 2023	BVL	13 (6, 7) 13 controls (3, 10), age and sex-matched	Patients: 63.5 years, mean 3–300 months Controls: 57.1 years	Decrease in bilateral para- hippocampal gyri
	Vestibular neuritis (VN)			
Hong et al., 2014	Unilateral VN	9 (6, 3) Patients served as own controls over time	Patients: mean, 49.2 years, <2 days and again at 3 months	Bilateral increase in gray matter volume
	Meniere’s Disease			
Van Cruijsen et al., 2007	MD	10 (6, 4) 10 controls (5, 5)	Patients: mean, 50.6 years, mean 9.9 years Controls: mean, 48.4 years	Left HPC atrophy
Seo et al., 2016	MD	38 (13, 25) 76 controls (26, 50)	Patients: mean, 49.2 years, mean 3.7 years Controls: mean, 49.1 years	Left hippocampal atrophy
Jian et al., 2023	Unilateral MD	99: early MD (50) (21, 29), late (49) (18, 31) 50 controls (16, 34) (16, 34)	Early: mean, 47.6 years, mean, 46.34 years Late: 51.23 years, mean, 71.25 years Controls: mean, 49.6	Decrease in late stage MD
	Persistent Perceptual Postural Dizziness (PPPD)			
Wurthmann et al., 2017	PPPD	42 (20, 22) 42 controls (20, 22)	Patients: mean, 39.28 years, mean 3.78 years Controls: mean, 37.97 years	Gray matter volume decrease
	Age-related vestibular dysfunction			
Kamil et al., 2018	age-related	103 (74, 29) Regression study, so age is the control	60 to >90 years	Lower volume related to lower cVEMP amplitude
Jacob et al., 2020	age-related	80 (64, 16) Regression study, so age is the control	Mean 77.5 years	Bilateral atrophy related to lower cVEMP amplitude
	Abnormal cVEMP			
Cohen et al., 2022	Abnormal cVEMP	22: Alzheimer’s disease or mild cognitive impairment (MCI)(14, 12) historical controls: 62 (21, 41)	Patients: mean, 77.8 years Controls: 75.8 years	Decrease in left HPC

UVL, unilateral vestibular loss; BVL, bilateral vestibular loss; MD, Meniere’s Disease; VN, vestibular neuritis; PPPD, persistent perceptual postural dizziness; cVEMP, cervical vestibular-evoked myogenic potential.

## Evidence for hippocampal volume changes following vestibular loss

To date, 16 studies (see Table 1) have examined HPC volume associated with some form of vestibular loss, mostly bilateral vestibular loss (BVL), but also unilateral vestibular loss (UVL), vestibular neuritis (VN), Meniere’s Disease (MD), Persistent Perceptual Postural Dizziness (PPPD) or age-related vestibular loss.

The first study was published by Brandt et al. (2005). They studied 10 patients who received bilateral vestibular neurectomies as a treatment for Neurofibromatosis Type 2 (NF2), some 5–10 years before the study. Only 1 patient had total post-operative hearing loss. Using a computerized virtual Morris Water Maze, they were shown to exhibit significant spatial memory deficits compared to age- and sex-matched controls. However, they also exhibited a bilateral atophy of the HPC of approximately 17%. Similar results have been reported in other studies, although often the HPC volume decreases have been more subregion-specific and stratified (e.g., Göttlich et al., 2016; Kremmyda et al., 2016; Schöne et al., 2022; Lee et al., 2023). Some studies of patients with BVL have reported no significant changes in HPC volume (e.g., Cutfield et al., 2014; Dordevic et al., 2021). Of course, if HPC atrophy did occur, its specific pattern might be likely to be related to the exact nature of the BVL, whether it was complete (e.g., Brandt et al., 2005) or partial (e.g., Göttlich et al., 2016; Kremmyda et al., 2016), the time that had elapsed since the loss of function, and even perhaps the sex of the patient (Smith P. et al., 2019).

Studies of patients with UVL have yielded somewhat different results. Hüfner et al. (2007) reported no changes in HPC volume in UVL patients. Similar results were reported by Dordevic et al. (2021). On the other hand, Schöne et al. (2022) reported a decrease in the volume of the right subiculum in UVL, but no change in total volume or in BVL patients (Table 1).

Only 3 studies have examined HPC volume in patients with MD. Van Cruijsen et al. (2007) found that MD was associated with an atrophy of the left HPC, as did Seo et al. (2016). On the other hand, Jian et al. (2023) reported that unilateral, late stage MD was associated with a bilateral HPC atrophy.

Wurthmann et al. (2017) reported a HPC gray matter volume decrease in patients with PPPD. Two studies of age-related vestibular dysfunction, quantified in terms of cervical vestibular-evoked myogenic potentials (cVEMPs), reflecting saccular function, have reported bilateral HPC atrophy related to lower cVEMP amplitude (Kamil et al., 2018; Jacob et al., 2020). Cohen et al. (2022) has also reported that abnormal cVEMPs in the elderly are associated with a decrease in left HPC volume.

Somewhat of an outlier is a study by Hong et al. (2014), which reported a bilateral *increase* in HPC gray matter volume between less than 2 days, and 3 months, following unilateral VN. However, this was a longitudinal study in which the patients served as their own controls; therefore, there was no independent healthy control group to indicate whether the patients initially exhibited HPC atrophy. Whether the increase in HPC volume over time was related to the specific nature of VN compared to other causes of UVL or BVL, is unknown.

Taken together, the effects of vestibular loss on HPC volume appear very complex and vary by specific vestibular disorder and even area of the HPC. Whether or not changes in HPC volume take place are likely to be affected also by the amount of time the patients had been suffering the vestibular disorder, its severity, whether there was concomitant hearing loss, and whether there was a history of other associated neurological or psychiatric disorders. The degree to which the patient copes with the vestibular dysfunction is likely to affect the levels of associated cortisol, which are known to affect HPC volume (Bremner et al., 2008; Brown et al., 2015; see Smith et al., 2021 for a recent review).

## Interpreting the meaning of changes in hippocampal volume following vestibular loss

Hippocampal volume has long been known to relate to cognitive function (e.g., Nedelska et al., 2012), and therefore it is natural to assume that HPC atrophy following vestibular loss – if it occurs – may be an explanation for the cognitive deficits that are observed.

However, there are three convincing arguments against this view: (1) Cognitive deficits have been observed in humans even in the absence of HPC atrophy; (2) HPC atrophy has not been reported in animal studies following vestibular loss, despite cognitive deficits; and (3) Animal studies have shown that the interruption of the transmission of vestibular information to the HPC has immediate consequences for HPC place cells, far too quickly to be explained by HPC atrophy.

Several studies have been published that have reported cognitive dysfunction associated with vestibular loss but without HPC atrophy. The most recent of these is Dordevic et al. (2021), who found spatial cognitive deficits in patients with UVL or BVL but without changes in HPC volume. Hüfner et al. (2007) reported that patients with right vestibular loss performed worse on spatial memory tests; however, there was no evidence of hippocampal atrophy. To the best of my knowledge, no study in humans with vestibular loss has reported HPC atrophy without some form of cognitive dysfunction. The only animal studies to have measured HPC volume following vestibular loss (BVL) have also found no significant change compared to controls, despite overwhelming evidence that BVL causes spatial cognitive deficits (Besnard et al., 2016). Besnard et al. (2012) used bilateral intratympanic injections of sodium arsanilate in rats and observed deficits in spatial memory without any change in HPC volume. Simular results were reported by Zheng et al. (2012) following bilateral surgical vestibular lesions, and this study included not only volume measurements but cell counts in HPC subregions. There is overwhelming evidence for spatial memory deficits in animals following UVL or BVL (see Besnard et al., 2016 for an extensive review), and yet it seems HPC atrophy is not necessary for them to develop. One possible explanation is that HPC neurogenesis, stimulated by the locomotor hyperactivity that tends to occur in rodents following BVL, somehow compensates for any atophy (see Smith, 2017 for a review). Nonetheless, some structural changes have been reported in the rat HPC following BVL. Balabhadrapatruni et al. (2016) reported an atrophy of dendrites in the CA1 region of the HPC at 14 months post-BVL.

**FIGURE 1:**
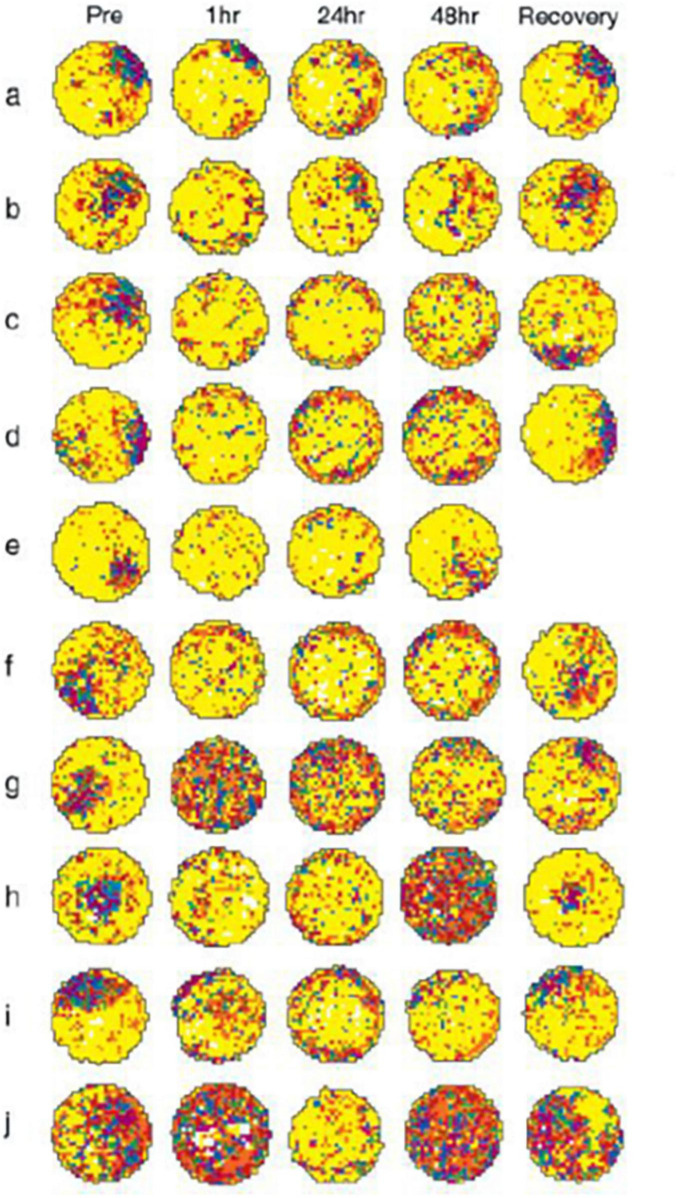
Vestibular inactivation disrupts location-specific firing in hippocampal place cells: examples of firing fields of all 10 cells recorded, before and after inactivation of the vestibular apparatus. a–j: For each map, increasing rates of discharge are coded from yellow, orange, red, green, blue, and purple, with yellow pixels depicting locations visited where no spikes were fired. Pixels that were never visited during the recording session are coded white. Each map was autoscaled such that the number of pixels in the next higher firing rate category was equal to 0.8 times the number of pixels in the lower firing rate category (Muller et al., 1987). For each example, pre depicts activity recorded during the baseline session; postinjection activity is depicted in the remaining plots under the headings 1 h, 24 h, 48 h, and Recovery. In each case, recovery represents that activity acquired during the recording session at which vestibular function was judged as restored. Respective recovery time points for each cell were as follows: a, 60 h; b, 72 h; c, 60 h; d, 72 h; e, 48 h; f, 72 h; g, 60 h; h, 96 h; i, 72 h; and j, 72 h. k: Unit waveform traces acquired during recording the cell depicted in c, at each of the time points before and after vestibular inactivation (i–v: Pre; Post 1 h; 24 h; 48 h; and Recovery). The calibration scale represents 50 μV/200 μs. l: Representative spike trace records depicting complex spike activity of the cell depicted in c, acquired at each of the time points before and after vestibular inactivation (i–v: Pre; Post 1 h; 24 h; 48 h; and Recovery). Calibration scale represents 50 μV/10 ms. From Stackman et al. (2002) with permission.

However, by far the most convincing evidence that HPC atrophy is not necessary for the cognitive deficits associated with vestibular loss, comes from the original HPC place cell study published by Stackman et al. (2002). They use bilateral intratympanic injections of tetrodotoxin (TTX) to reversibly inactivate the rat peripheral vestibular system. What they found was very important. Place cell responses in behaving rats deteriorated and fragmented compared to control animals; however, they started to do so within 1 h of the injections (see Figure 1). Since it is very unlikely that the HPC could start to atrophy within 1 h, this result strongly suggests that it is the loss of vestibular input to the HPC that causes the spatial cognitive deficits and not any longer term structural changes. Russell et al. (2003) reported similar results for HPC place cells following BVL, but they used surgical lesions and their recordings did not begin until 6 weeks post-op. Short-term reductions in the power of HPC theta rhythm have also been found following bilateral intratympanic injections of sodium arsanilate (Tai et al., 2012). Similar results were reported for entorhinal cortex theta rhythm following bilateral intratympanic injections of TTX (Jacob et al., 2014). However, one caveat with these studies in rats, in terms of their relevance to vestibular loss in humans, is that they have all employed intratympanic injections of TTX, which potentially affected the auditory system as well. Since humans do not normally experience vestibular loss through such a process, this separates these studies from human clinical studies. Nonetheless, taken together, the results of these animal studies suggest that HPC atrophy may not be necessary for the dysfunction of HPC place cells and theta rhythm following vestibular loss and that it may be the immediate loss of the transmission of vestibular information that is responsible for both HPC dysfunction and spatial memory deficits.

## Discussion

Many studies have now been published reporting the results of HPC volume measurements following various kinds of vestibular loss. Most of them have reported some volume changes, usually decreases, at least in circumscribed areas of the HPC; however, some of them have not (see Table 1). Over 2 decades, the finding of HPC atrophy following vestibular loss has often been interpreted as a potential explanation of the spatial cognitive deficits that are usually observed in patients with vestibular disorders (see Smith, 2022a,b for a recent review). This is a natural assumption since HPC atrophy, in general, has been associated with cognitive deficits (e.g., Nedelska et al., 2012). However, the results of HPC studies in animals, in which vestibular information has been interrupted suddenly, and in some cases reversibly (e.g., Stackman et al., 2002), suggest that slow structural changes such as HPC atrophy are not necessary for place cell or theta rhythm dysfunction, and therefore are probably not necessary for the spatial cognitive deficits observed.

It is noteworthy that some of the studies reporting HPC atrophy following vestibular loss involve patients who lost vestibular function 5–10 years before testing (e.g., Brandt et al., 2005). During this time, many concomitant changes may have taken place, such as the development of affective disorders like anxiety disorders and/or depression, since they are often associated with vestibular dysfunction (see Staab, 2019 for a review). Chronic stress resulting in high circulating levels of corticosteroids is known to be associated with HPC atrophy (Bremner et al., 2008; Brown et al., 2015; see Smith et al., 2021 for a recent review). and it is possible that the consequences of living with a vestibular disorder partly contribute to any HPC atrophy observed.

It is also worth noting that vestibular information appears to be transmitted to the HPC through highly complex pathways contributing to different aspects of HPC function (e.g., spatial memory versus emotion) (Bannerman et al., 2004; Hüfner et al., 2011; Hitier et al., 2021) and therefore for many vestibular disorders, it seems likely that any structural changes in the HPC would usually be stratified rather than global. In conclusion, I suggest that the spatial cognitive deficits observed in patients with vestibular loss are more likely to be caused by the rapidly occurring effects of the loss of transmission of vestibular information to the HPC rather than long-term structural changes in the HPC. This, of course, does not rule out the possibility that HPC atrophy, when it occurs, causes other, longer term effects.

## Data availability statement

The original contributions presented in this study are included in the article/supplementary material, further inquiries can be directed to the corresponding author.

## Author contributions

PS: Conceptualization, Writing – original draft, and Writing – review & editing.
